# Transition Metal Transport in Plants and Associated Endosymbionts: Arbuscular Mycorrhizal Fungi and Rhizobia

**DOI:** 10.3389/fpls.2016.01088

**Published:** 2016-07-29

**Authors:** Manuel González-Guerrero, Viviana Escudero, Ángela Saéz, Manuel Tejada-Jiménez

**Affiliations:** Centro de Biotecnología y Genómica de Plantas, Universidad Politécnica de Madrid (UPM) – Instituto Nacional de Investigación y Tecnología Agraria y Alimentaria (INIA)Madrid, Spain

**Keywords:** metal homeostasis, plant–microbe interaction, arbuscular mycorrhiza, rhizobia, transition metals

## Abstract

Transition metals such as iron, copper, zinc, or molybdenum are essential nutrients for plants. These elements are involved in almost every biological process, including photosynthesis, tolerance to biotic and abiotic stress, or symbiotic nitrogen fixation. However, plants often grow in soils with limiting metallic oligonutrient bioavailability. Consequently, to ensure the proper metal levels, plants have developed a complex metal uptake and distribution system, that not only involves the plant itself, but also its associated microorganisms. These microorganisms can simply increase metal solubility in soils and making them more accessible to the host plant, as well as induce the plant metal deficiency response, or directly deliver transition elements to cortical cells. Other, instead of providing metals, can act as metal sinks, such as endosymbiotic rhizobia in legume nodules that requires relatively large amounts to carry out nitrogen fixation. In this review, we propose to do an overview of metal transport mechanisms in the plant–microbe system, emphasizing the role of arbuscular mycorrhizal fungi and endosymbiotic rhizobia.

## Introduction

Iron, copper, zinc, and some other transition metals are essential nutrients for plants (Frausto da Silva and Williams, 2001; Clemens et al., 2002; Morrissey and Guerinot, 2009; Pilon, 2011; Olsen and Palmgren, 2014). It is estimated that a third of the proteins of a typical cell are metalloproteins (Dupont et al., 2006; Finkelstein, 2009), which participate in almost every biological process, from photosynthesis to seed production. However, plants often have to live in soils with low metal bioavailability (Alloway, 2008; White and Broadley, 2009). This prevalent metal deficiency limits plant growth and tolerance to stress, lowers yields, and reduces crop nutritional value. Consequently, human diet in many areas of the world does not include the minimum metal nutrient requirements, causing from minor immunological alterations to death (Grotz and Guerinot, 2006; Jamison et al., 2006; Alloway, 2008; White and Broadley, 2009; Akkermans et al., 2016). As a result, considerable effort has been made to increase plant metal uptake by using metal fertilizers (Larbi et al., 2010; Wei et al., 2012; López-Rayo et al., 2015), and developing new crop varieties with improved metal uptake capabilities (Hirschi, 2009; Masuda et al., 2012; Eggert and von Wiren, 2013).

However, when aiming to improve plant nutrition in a sustainable manner, the role of plant-associated microorganisms, the plant microbiome, should also be contemplated (East, 2013). Within a plant, different compartments can be identified, each with a different microbial community, such as the phyllosphere (leaves), the rhizosphere (the portion of soil directly affected by plant exudates), or the endosphere (the internal tissues of the plant). For almost a 100 years, since Hiltner’s pioneer work (Hiltner, 1904), the composition and role of the plant microbiome has been studied. These efforts have accelerated in recent years following the improvement of deep sequencing technologies and bioinformatics pipelines to process the vast amount of data obtained (Scholz et al., 2012). All these efforts have revealed that plants rely on their microbiome for a wide range of processes: from resisting pests to colonizing new environments (Haney and Ausubel, 2015; Santhanam et al., 2015; Vandenkoornhuyse et al., 2015). Arguably the most important role of the plant microbiome is to improve nutrient uptake (Li et al., 1991; Smith and Smith, 2011; Rana et al., 2012; Udvardi and Poole, 2013; Hart et al., 2014). Plant–microbe interactions enabled plant colonization of terrestrial environments by allowing the recovery of nutrients from soil (Field et al., 2015). Many of these symbiotic relationships, established in the early stages of plant evolution, have been maintained to date. As a result, we should study the plant-microbe holobiont (the host plant plus its associated microbiome) rather than the isolated plants, if we are to consider plant nutrient uptake in natural environments (Bordenstein and Theis, 2015; Vandenkoornhuyse et al., 2015).

This review intends to approach plant transition metal nutrition, contemplating how plants incorporate metals from soil either on their own or assisted by associated microorganisms (mainly arbuscular mycorrhiza), and how they are delivered to leaves, seeds, and, in the case of legumes, to endosymbiotic bacteria living within the root nodule cells. To do this, first we will describe some of the main protein families involved in metal transport and intracellular metal trafficking to have a conceptual frame to place the plethora of proteins involved in metal plant and plant-endoymbiont metal trafficking. For information on how plants protect themselves against toxic metal concentrations, please consider Clemens and Ma (2016), or Sharma et al. (2016) as two of the most recent reviews on the topic.

## Proteins and Small Organic Molecules Involved in Transition Metal Transport

**FIGURE 1:**
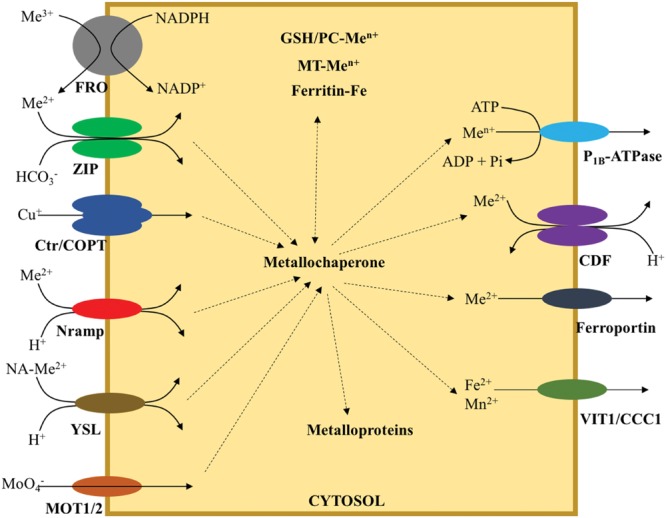
**Main systems involved in metal transport and intracellular trafficking.** Co-transported ion is indicated where known. Dotted arrows indicate exchange of metal ligands.

Cellular metal homeostasis requires a highly precise regulation to ensure that transition elements are kept at high enough levels to carry out their biological functions, but not so high that they can catalyze the production of free radicals, or displace less abundant elements in the core of metalloproteins. This balance is achieved by the coordinated action of ferroreductases that provide the metals in the correct oxidative state, together with the transporters that move them across membranes, the small soluble metal binding molecules and proteins that ferry metals among the different transporters and to apoproteins, and metal detoxifying molecules that buffer the cell against a sudden increase in transition metal levels (**Figure 1**). In this section, we will describe some of the key aspects of the different molecules involved in transition metal transport.

### Ferroreductases

In the case of iron and copper are not translocated as a metal-chelator complex, the substrate is the reduced form (Fe^2+^/Cu^+^), rather than the more prevalent in aerobic environments oxidized state (Fe^3+^/Cu^2+^). Therefore, a mechanism must exist to reduce Fe^3+^/Cu^2+^. This process is carried out by members of the ferroreductase oxidase (FRO) family (Robinson et al., 1999; Connolly, 2014). FROs are membrane proteins with eight transmembrane domains and a large soluble domain with bound NADPH and FAD, in addition to an oxidoreductase motive (Connolly, 2014). Reducing electrons are provided by NADPH and directed toward the metals in a process that very likely requires two intramembrane heme groups.

### Transition Metal Transporters

Once the metals are in the proper oxidative state or bound to the correct metallophore, they will be transported across the membrane by the different transition metal transporters. There are several families of them with distinct metal affinities and direction of transport. For the purposes of this review, we will classify them based on their direction of transport (into or out of the cytosol) since this will define their role in metal trafficking from soil to sink organs.

#### Transporters that Introduce Metals into the Cytosol

The best studied families are the ZIP, Ctr/COPT, Nramp, YSL, and MOT families. Their key features are:

• ZIP transporters (Zinc resistance transporter, Iron-resistance transporter-like Proteins). These are a ubiquitous family of divalent metals transporters (mainly Fe^2+^, Zn^2+^, Ni^2+^, and Mn^2+^; Eide et al., 1996; Grotz et al., 1998; Guerinot, 2000; Mizuno et al., 2005). Although no structure is available of any ZIP transporter, computational modeling indicates that they act as homodimer, where each monomer has eight transmembrane domains (Antala et al., 2015). ZIP transporters contain a conserved cytosolic histidine-rich loop between transmembrane (TM) domains 3 and 4 in eukaryotes (Taylor and Nicholson, 2003), that seems to be responsible for metal specificity and transport rate (Antala et al., 2015). The energetics of transport has not been clearly defined yet, with authors proposing a HCO_3_^-^ symport (Gaither and Eide, 2000; Liu et al., 2008), while others suggest that they work as channels (Lin et al., 2010). Members from this family include the transporters responsible for iron and zinc uptake from soil (Eide et al., 1996; Lin et al., 2009).
• Ctr/COPT transporters (Copper transporter). They have only been found in eukaryotes, being known as COPT transporters in plants (Sancenón et al., 2003) and Ctr in animals and fungi (Kim et al., 2013). They work as homo- or heterotrimer of a three-transmembrane domain (Zhou and Thiele, 2001; Aller and Unger, 2006; Nose et al., 2006; De Feo et al., 2010), forming a channel responsible for specific Cu^+^ transport (Dancis et al., 1994; Eisses et al., 2005; Sinani et al., 2007). In plants, COPT proteins have been suggested to play a role in copper uptake from soil and delivery to pollen (Sancenón et al., 2004).
• Nramp transporters (Natural Resistance-Associated Macro phage Protein). This family of transporters can be found in the three domains of life (Nevo and Nelson, 2006). They are a monomeric protein spanning 11 transmembrane domains (Ehrnstorfer et al., 2014). Transport is driven by a H^+^ symport (Gunshin et al., 1997). Nramp transporters have a wide range of metal substrates, typically Fe^2+^, Mn^2+^, Co^2+^, and Zn^2+^ (Nevo and Nelson, 2006). Metal binding sites are integrated by a carbonyl from a peptide bond in TM6, a Met in the same domain and two Asp from TM1, in a planar geometry (Ehrnstorfer et al., 2014). Some Nramp transporters have been proposed to be involved in iron and manganese uptake by the root epidermis (Cailliatte et al., 2010).
• YSL transporters (Yellow Stripe-like proteins). *Yellow stripe* is a phenotype identified in maize where intervenal chlorotic (yellow) zones are observed (Beadle, 1929). This chlorosis is caused by deficient iron uptake, the result of the mutation of a root epidermal transporter (*YS1*; Beadle, 1929; Curie et al., 2001). Members of the YSL family can only be found in plants (Curie et al., 2008), although YSL belong to the larger OPT (oligopeptide transporter) family that is also present in fungi (Lubkowitz, 2011). YSLs transporters do not use free metals as substrate, but a complex of metals with nicotianamine (NA) or its derivatives (Curie et al., 2008). NA is a non-proteogenic amino acid that is synthesized from *S*-adenosyl-methionine by the enzyme NA synthase (NAS; Higuchi et al., 1999). Transport by YSL proteins is energized by a H^+^-symport (Schaaf et al., 2004). Additionally, at least some of the plant OPT transporters have also been associated by metal transport (Lubkowitz, 2011; Zhai et al., 2014; Bashir et al., 2015), although the identity of the metal complex transported still remains elusive. Not much is known about the structure of these proteins, with different models proposing a range of 11–16 transmembrane regions (Lubkowitz, 2011). In broad terms, YSL transporters are involved in metal uptake from soil in monocots and in long-distance metal distribution in both monocots and dicots (Conte and Walker, 2011).
• MOT1 (Molybdate transporter type 1). In contrast to other transition metals, molybdenum is transported as the oxoanion molybdate. These transporters were first identified in parallel in *Chlamydomonas reinhardtii* and in *A. thaliana* and show high affinity for molybdate (Tejada-Jiménez et al., 2007; Tomatsu et al., 2007). They have been associated with Mo uptake in *C. reinhardtii*, while in *A. thaliana* its specific role still remains elusive since some authors suggest a role in molybdate uptake from soil, while other point to a role in mitochondrial molybdenum homeostasis (Tomatsu et al., 2007; Baxter et al., 2008). Other members of the family would be involved in molybdate storage. Aside from the MOT1 family, an additional family, MOT2, has been identified also involved in molybdate uptake in *C. reinhardtii* (Tejada-Jiménez et al., 2011). MOT2 family members role in higher plants has not been determined yet.

#### Transporters that Remove Metals Out of the Cytosol

The best studied families are the P_1b_-ATPases, CDF, ferroportins, and VIT/CCC1 families. Their key features are:

• P_1b_-ATPases. They are a clade of the P-type superfamily of ATPases (which also includes the Na^+^/K^+^-ATPase, or the H^+^-ATPase; Axelsen and Palmgren, 1998). This clade is conserved in all three domains of life and is subdivided in subclades with different metal specificities (P_1b-1_ for Cu^+^, P_1b-2_ for Zn^2+^,…) (Argüello, 2003; Argüello et al., 2007). The transporter is a monomer with several transmembrane spanning domains (from 6 to 8). The last two cytosolic loops are enlarged and comprise the ATPase domain (closest to C-terminus) that drives transport, and the activator domain. Metal specificity of each subclade is determined by specific amino acids in the three transmembrane helices closest to C-terminus (Argüello, 2003; Argüello et al., 2007; González-Guerrero et al., 2008a; Raimunda et al., 2012). In addition, they frequently have cytosolic metal binding domains in N and/or C-termini with a regulatory function (Mandal and Argüello, 2003; Eren et al., 2007; González-Guerrero and Argüello, 2008). P1b-ATPases are involved in long-distance Cu^+^ and Zn^2+^ transport in plants, as well as metal transport into organnelle (Hussain et al., 2004; Andrés-Colás et al., 2006).
• CDF transporters (Cation Diffusion Facilitator). Members of this family are present in all organisms (Kolaj-Robin et al., 2015). Their substrate are divalent metals such as Fe^2+^, Zn^2+^, or Mn^2+^, coupled to a H^+^ antiport (MacDiarmid et al., 2002; Rubio-Sanz et al., 2013; Gupta et al., 2014; Raimunda and Elso-Berberián, 2014). The functional transporter is a homodimer (Lu and Fu, 2007). The monomer has six transmembrane domains with a His-rich region in the cytosol between the fourth and the fifth transmembrane region, which is only present in eukaryotic CDF transporters (Kolaj-Robin et al., 2015). There are three metal binding domains in the protein: site I in the transmembrane region, site II in the membrane-cytosol interface and site III in the C- terminal domain, but only I and III seem to be directly involved in transport (Lu and Fu, 2007). Site I very likely defines the metal to be transported, which is coordinated by two Asp in TM2 and a His and Asp in TM5 in the Zn transporter YiiP. Site III facilitates dimerization, and would consequently, have a regulatory mechanism on metal transport. Most plant CDFs, known as MTPs, have been associated to metal detoxification, although others could play a role in long-distance metal transport (Ricachenevsky et al., 2013).
• Ferroportins. They are only present in eukaryotes (Muckenthaler et al., 2008; Morrissey et al., 2009). The topology is predicted to comprise 11 transmembrane domains with a large extracytosolic loop between TM5 and 6 (Yeh et al., 2011). The metal substrates are Fe^2+^, Ni^2+^, and Co^2+^ (Schaaf et al., 2006; Muckenthaler et al., 2008; Morrissey et al., 2009), that are extruded out of the cytosol by a yet-to-be-determined mechanism. Ferroportins would be involved in iron/cobalt uploading of the xylem (Morrissey et al., 2009).
• VIT1/CCC1. Fe^2+^ and Mn^2+^ has been identified as substrate (Li et al., 2001; Kim et al., 2006). Very little is known on their structure and transport mechanism, other than it is predicted to cross the membrane five times (Kim et al., 2006).

### Soluble Metal Carriers

Unlike alkali or alkali-earth metals, transition elements are not kept “free,” hydrated, in the cytosol (O’Halloran and Culotta, 2000). This means that if they are not transported as metal-conjugates, they would have to bind a protein (metallochaperone) or to some small organic molecule as they are released by the metal transporter. It is estimated that at least a third of the metalloproteins of the cell obtain their metal cofactor from a metallochaperone (Foster et al., 2014). This has been clearly shown for copper, where several different proteins have been proposed to mediate metal delivery from the Ctr/COPT transporters to P-type ATPases or to apoproteins (O’Halloran and Culotta, 2000). Similar elements have been identified for Ni, clusters Fe-S, and molybdenum (Vergnes et al., 2006; Chan Chung and Zamble, 2011; Mapolelo et al., 2013). These observations indicate that at least in these cases, transition metal transport and delivery is mediated by specific protein–protein interactions, rather than by simple metal affinities and mass equilibria. However, for some other metals, specially for zinc, a labile metal pool has been proposed (Atkinson et al., 2010). Nevertheless, in these cases, transition metals are not free, hydrated, but bound to small organic molecules such as amino acids and organic acids (Sinclair and Krämer, 2012; Ma et al., 2014). In plants, some of these metal complexes are responsible for metal delivery by the vasculature and across simplastically disconnected tissues (Roschzttardtz et al., 2011; Álvarez-Fernández et al., 2014).

### Metal Concentration Buffers/Metal Storage Proteins

When cytosolic metal concentrations rise above a certain level, metal binding capabilities of metallochaperones and organic molecules are overloaded. In that case, excessive metal levels have to be buffered by other means, since they could become toxic. Metallothioneins seem to be an universal solution to this problem (Blindauer and Leszczyszyn, 2010), since they are present in all three domains of life. These are cysteine-rich proteins that show high affinity for copper, zinc, or cadmium. Metallothioneins are kept in the cytosol. They have been predicted to have other functions based on the high number of cysteines, such as using internal disulphide bridges to control the oxidative state of the cell (Maret, 2011). Similarly participating in metal control in the cell is glutathione (GSH; Helbig et al., 2008), also based on the cysteine thiol in the tripeptide. To increase metal binding capabilities, plants and some fungi synthesize oligomers of GSH with the enzyme phytochelatin (PC) synthase (Ha et al., 1999). This enzyme uses the energy released by removing the peptide bond between Cys and Gly to condensate the γ-Glu-Cys dipeptide to the Glu of GSH. The resulting oligomer (typically of 2–10 units) is known as PC and can bind copper, cadmium, and zinc with high affinity in the cytosol (Cheng et al., 2005). Excess PC-metal complexes can be stored in vacuoles after being transported by ABC transporters (Ortiz et al., 1995; Song et al., 2010b).

The vacuole can also store other metals or other metal species. For instance, Zn-NA complexes are used to maintain cytosolic zinc homeostasis (Haydon et al., 2012). In the case of iron, the main reservoir are the vacuoles (Roschzttardtz et al., 2009; Lanquar et al., 2010), and the plastids (Terry and Abadía, 1986). In the later, iron is densely packed, in a quasi-crystalline structure by ferritins, oligomeric proteins that form a protein shell within which iron is stored (Briat et al., 2010).

## Transition Metal Uptake From Soil

**FIGURE 2:**
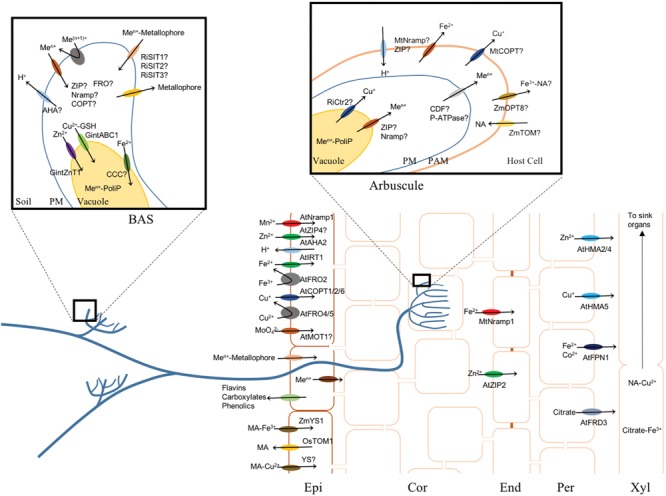
**Diagram of the main transport processes in roots, using Strategy I or Strategy II or the mycorrhizal pathway (left inset for uptake by Branched Absorbing Structures, right inset for metal delivery by arbuscules).** At is used as a prefix to indicate *A. thaliana* proteins, Os for *Oryza sativa*, Zm for *Zea mays*, Mt for *M. truncatula*, Gint for *Glomus intraradices*, Ri for *Rhizophagus irregularis*, and AMF for arbuscular mycorrhiza. Epi stands for Epidermis, Cor for Cortex, End for Endodermis, Per for Pericycle, Xyl for Xylem, PM for Plasma Membrane, PAM for Periarbuscular Membrane, and Me^n+^ for a general metal.

Transition metals are tightly bound to soil particles and have low solubility, specially in basic soils (Ruel and Bouis, 1998; Grotz and Guerinot, 2006). As a result, a fierce competition for these nutrients is established in the rhizosphere (Lugtenberg and Kamilova, 2009), where metal uptake efficiency is critical for proliferation. Plants have adapted to this environment using different and complementary strategies (**Figure 2**): (i) Using the metal solubilized by rhizospheric microorganisms, (ii) directly increasing metal solubility in their rhizosphere, and (iii) using mycorrhizal fungi to mine a wider soil area for metals.

### Rhizospheric Microorganisms and Plant Metal Uptake

Bacteria can use both a reductive and a chelating strategy for metal uptake (Blindauer, 2008; Cornelis, 2010). In the chelating strategy, a wide array of metallophores such as enterobactin, pyoverdin, or anguibactin, are secreted and then, the metal-chelator complex is introduced though different metal transport complexes (Crosa, 1989; Bellenger et al., 2008; Kraepiel et al., 2009). The presence of these metal-chelator transporters does not necessarily indicate the ability of that particular bacteria of synthesizing the chelator. It is not uncommon for bacteria to be able to “steal” some of the metal complexes formed with chelators synthesized by other bacteria (Jurkevitch et al., 1992; Loper and Henkels, 1999; Cornelis and Matthijs, 2002).

Plants benefit from this scenario. As bacteria acidify the surrounding soil, some of the solubilized metal can be used by the plant (Zhang et al., 2009; Marschner et al., 2011). It can also use bacterial metallophores as sources of metals (Bar-Ness et al., 1992; Vansuyt et al., 2007). In other cases, root architecture is altered by rhizospheric bacteria increasing its exploratory capacity, and consequently, more access to nutrients is obtained (López-Bucio et al., 2007; Zamioudis et al., 2013). Rhizospheric bacteria can also directly affect plant metal uptake mechanism by means of volatile organic compounds (Zhang et al., 2009; Orozco-Mosqueda et al., 2013; Zamioudis et al., 2015). These molecules are able to induce the iron deficiency response in a *FIT1*-dependent manner (Zhang et al., 2009; Zamioudis et al., 2015), leading to upregulation of ferroreductase activity in *A. thaliana* roots as well as of iron mobilizing phenolics (Zamioudis et al., 2014, 2015). These volatiles are detected by the shoots, triggering the transmission of systemic iron deficiency signals (Zamioudis et al., 2015).

The relationship between plant and the microorganisms in its rhizosphere is dynamic. The plant microbiome can be altered to adapt to different developmental stages or to biotic and abiotic stresses (Carvalhais et al., 2015). This is achieved by changing the composition of root exudates (Chaparro et al., 2013; Liang et al., 2013). Similarly, metal deficiency also results in changes in the composition of root exudates (Kobayashi and Nishizawa, 2012; Sisó-Terraza et al., 2015). These changes could also be responsible for the variations observed in the plant microbiome of plants grown in metal deficient soils (Pii et al., 2016).

### Transition Metal Uptake by the Root Epidermis

Plants have developed a number of mechanisms to obtain these nutrients directly from soil. This has been best studied in the case of iron uptake, where two approaches (Strategies I and II) are followed (Kobayashi and Nishizawa, 2012) (**Figure 2**). In Strategy I, plants lower the pH of the surrounding soil by extruding H^+^ by root epidermal H^+^-ATPases. Acidifying soils increases Fe^3+^ solubility and this is further increased by reducing it to Fe^2+^ with epidermal FRO proteins. Then, Fe^2+^ is transported into the cell by ZIP and Nramp transporters. In *A. thaliana*, the proteins carrying out these roles are mainly AHA2 (Santi and Schmidt, 2009), FRO2 (Robinson et al., 1999), and IRT1 (Eide et al., 1996). This process is very tightly controlled to prevent iron overload of the plant using at least three levels of regulation: transcriptional, posttranslational, and intracellular trafficking (Kobayashi and Nishizawa, 2012). At the transcriptional level, a number of bHLH factors control the expression of these genes (Colangelo and Guerinot, 2004; Sivitz et al., 2012). Ubiquitinization plays a role in controlling the protein levels of some of these transcription factors, as well as membrane recycling of IRT1 (Barberon et al., 2011). Furthermore, upon reduction of non-iron metal substrates of IRT1, this transporter polarly localizes to the soil-facing side of the epidermal plasma membrane (Barberon et al., 2014). Strategy I seems to be the most ancient one, since it is used by dicots and some monocots (rice; Ricachenevsky and Sperotto, 2014).

In Strategy II, carried out by monocots, iron is not transported as Fe^2+^, but as a complex with mugineic acids (MA; Kobayashi and Nishizawa, 2012), a NA-derivative with high affinity for Fe^3+^. Subsequently the complex is introduced into the plant by YSL transporters (Curie et al., 2001). For transport to occur, MAs have to be extruded to the rhizosphere mediated by TOM1-like transporters (Nozoye et al., 2011). This process is also under transcriptional control, being IDEF1, IDEF2, and IRO2 some of the key transcription factors involved (Ogo et al., 2006, 2008; Kobayashi et al., 2007). Strategy II is carried out by all monocots (Ricachenevsky and Sperotto, 2014).

However, the separation of the two strategies (reductive vs. chelating) is not as straightforward as originally thought. Many Strategy I plants release in their root exudates a number of molecules that can solubilize Fe^3+^ and form complexes that are subsequently introduced into the plant. These include phenolics, coumarins carboxylates, and flavins (Cesco et al., 2010; Fourcroy et al., 2013; Schmidt et al., 2014; Valentinuzzi et al., 2015). In addition, the flavins could also contribute to Fe^3+^ solubility by reducing it to Fe^2+^ (Sisó-Terraza et al., 2015).

Copper uptake from soil very likely follows similar strategies as for iron. In *Arabidopsis*, two FRO proteins, FRO4 and FRO5, are strong candidates for reducing Cu^2+^ to Cu^+^ (Bernal et al., 2012), which would be introduced into the plant via COPT transporters (candidates are COPT1, 2, or 6; Gayomba et al., 2013). Furthermore, Cu^2+^ can bind MA precursor NA and be transported by YSL proteins (DiDonato et al., 2004), supporting the existence of a Strategy II approach of copper uptake. Other elements that would not suffer oxidation changes under physiological conditions (Zn^2+^, Mn^2+^, …) would be transported by ZIP and Nramp transporters present in the plasma membrane of epidermal roots (Lin et al., 2009; Cailliatte et al., 2010).

Molybdenum is incorporated by plants as molybdate, instead of a cationic form. In green algae *C. reinhardtii* this is done by two molybdate transporters belonging to the MOT1 and the MOT2 families corresponding to a high affinity (7 nM) and low affinity (550 nM) system, respectively (Tejada-Jiménez et al., 2007, 2011). Higher plant genomes also encode for MOT1 and MOT2 orthologs. In *A. thaliana* AtMOT1 seems to be required for molybdate uptake from soil, either if this is done directly or indirectly still remains to be defined, since conflicting data has been reported of its membrane localization (Tomatsu et al., 2007; Baxter et al., 2008).

### Transition Metal Uptake by Mycorrhizal Plants

Almost 90% of plants are able to establish an endosymbiotic relationship with fungi through their roots: the mycorrhiza (Smith and Read, 2008). Some of these fungi are basidiomycetes and ascomycetes that can establish a plethora of different types of mycorrhizal relationships: ectomycorrhiza, ectendomycorrhiza, arbutoid, orchioid, etc. (Peterson et al., 2004). However, the most common mycorrhiza (almost 85% of plants) is the arbuscular mycorrhiza established with fungi from the Glomeromycota phylum. Arbuscular mycorrhiza is formed through a complex and very regulated process (Gutjahr and Parniske, 2013). After spore germination, and detection of strigolactones, the germinating hyphae branches to maximize the chances of contacting the root epidermis. There, the fungus forms an appresorium that will allow penetration into the root cortex. Once in the cortex, the hyphae disperse though the intercellular spaces and at regular intervals, they penetrate into the cells, branching multiple times, and constituting an arbuscule. The arbuscules do not cross the plant cell plasma membrane, but establish a very close interface with a differentiated host plasma membrane cell, the periarbuscular membrane (Gutjahr and Parniske, 2013). Across these two membranes (arbuscular plasma membrane and periarbuscular membrane) nutrients are exchanged (Rausch et al., 2001; Javot et al., 2007). In addition, as the fungal intracellular mycelium and arbuscules develop, the fungal extracellular mycelium grows, producing branched absorbing structures (BAS) at regular intervals, which, when the conditions are right, they develop spores (Bago et al., 1998). In spite of sometimes reaching an area measured in square kilometers, the fungal colony is just one protoplasm, with millions of nuclei distributed at regular intervals in the coenocytic mycelium (Smith and Read, 2008). In natural environments plants greatly rely on arbuscular mycorrhizal fungi (AMF) to feed themselves (Smith and Read, 2008). It is estimated that they can transfer to their host over 90% of the phosphate, and over 50% of the fixed nitrogen in exchange of photosynthates (Smith and Smith, 2011). This nutrient exchange is critical for the symbiosis; otherwise the arbuscules are aborted (Javot et al., 2007; Helber et al., 2011; Breuillin-Sessoms et al., 2015).

The connection of AMF with plant transition metal nutrition has been known from very early on. Mosse (1957) showed that iron and copper content in apple seedlings increased upon mycorrhization. Further studies have shown a role of AMF in improving uptake of additional metals in several different plant species (Caris et al., 1998; Clark and Zeto, 2000; Cavagnaro, 2008). Experiments using radio-labeled metals and a mesh that created a fungal compartment, so that labeled metals could only be reached by fungal hyphae, proved that the host plant is able to recover metal from the soil through the mycorrhizal fungi (Caris et al., 1998). Further support for the existence of a mycorrhizal metal delivery pathway is that mycorrhizal plants diminish the expression levels of some root metal transporters compared to non-mycorrhizal roots. For instance, *M. truncatula* down-regulates a cortical ZIP protein upon mycorrhization (Burleigh et al., 2003). The specific contribution of the mycorrhizal pathway to plant metal nutrition has not been clearly determined, ranging from 20 to 50% (Ortas, 2012; Lehmann et al., 2014), although will greatly depend on soil type, fungal inoculum, and host. In tomato, for instance, zinc content of fruit was 50% higher in mycorrhizal plants (Cavagnaro et al., 2006). Metal transfer from AMF to their hosts is controlled to prevent metal overload of the host. For instance, when metal concentrations in soil are toxic, mycorrhizal plants accumulate less metals in their shoots than non-mycorrhizal ones (Diaz et al., 1996; Chen et al., 2003; Watts-Williams et al., 2013). Therefore, AMF act as metal buffers, increasing metal delivery to the plant under low metal levels, but decreasing metal uptake when toxic levels of metals are present.

Metal uptake by mycorrhizal fungi should be quite similar to free living fungi. Genomic evidence indicates that model AMF *Rhizophagus irregularis* genome encodes genes that are putatively involved in metallophore-metal uptake (*RiSIT1, RiSIT2*, and *RiSIT3*; **Figure 2**), as well as other that could be involved in metallophore syntheses (Tamayo et al., 2014). In addition, AMF genome encodes several different members of the Nramp, Ctr, and ZIP families of metal transporters (Tamayo et al., 2014), some of which could conceivably be involved in metal uptake from the soil, as it happens in other fungi (Dancis et al., 1994; Zhao and Eide, 1996; Cohen et al., 2000). Detailed expression and localization analyses of these genes is required to confirm this possibility.

The mechanism of nutrient delivery from AMF to the host is hypothesized to be mediated by vacuoles that act as carriers (Jin et al., 2005; Javot et al., 2006; González-Guerrero et al., 2008b), since a model based on nutrient diffusion would be too expensive given the energy required to establish gradients in the relative large distance from the extrarradical mycelium to the arbuscules. Vacuoles can be directed to different locations in an active way through the cytoskeleton (Allaway and Ashford, 2001). Phosphate that is incorporated by the BAS is delivered to the vacuoles where it is polymerized into polyphosphate fibers (Ezawa et al., 2004), that have a high density of negative charges. Other nutrients are loaded into the negatively charged vacuoles, helping to stabilize the charges (Jin et al., 2005; González-Guerrero et al., 2008b). This model for metal delivery to the host plant is supported in metal localization studies that show colocalization of phosphate and metals in vacuolar compartments (González-Guerrero et al., 2008b). Vacuoles would also play a role in metal detoxification. Vacuolar metal uploading has to be mediated by metal transporters. Two candidates are likely participating in this process: *GintZnT1* and *GintABC1*. The former is a CDF family member that can transport Zn^2+^ out of the cytosol, that is able to reduce cytosolic zinc levels when expressed in yeast (González-Guerrero et al., 2005). It seems to be involved in early response to Zn^2+^ increase in the cytosol, as indicated by the upregulation of its expression in the early moments of exposure to moderate zinc concentrations. *GintABC1* is an ABC transporter that is upregulated by increased copper, cadmium, and oxidative stress levels, being expressed at similar levels in the intraradical and extraradical mycelium (González-Guerrero et al., 2010). In addition, homologs of vacuolar iron transporter CCC1 and P-type ATPases have been identified in AMF genomes (Tamayo et al., 2014), being putative candidates for iron and copper vacuolar loading, respectively. All these genes could be responsible for metal loading of vacuoles. Under high metal concentrations, vacuoles would be diverted toward the spores (González-Guerrero et al., 2008b), explaining the protective effect that mycorrhiza has against toxic levels of metals in soils (Diaz et al., 1996; Ferrol et al., 2009).

Once the metal-loaded vacuole reaches the arbuscule, polyphosphate is hydrolized and then transferred to the host. As a result, the associated metals would be released into the cytosol by yet-to-be-determined metal transporters. Candidates for metal release are transporters of the Nramp, ZIP, and COPT families (**Figure 2**). Recently Tamayo et al. (2014) have proposed that *RiCtr2*, one of the three COPT transporters encoded in *R. iregularis* genome, could be moving Cu^+^ from the vacuole into the cytosol. This protein has the closest homology to a yeast vacuolar Ctr protein doing a similar function. Furthermore, it is expressed at the highest levels in the intraradical mycelium, where Cu^+^ would be released from the vacuole. However, further evidences (subcellular localization of the transporter, gene silencing by Host Induced Gene Silencing) are required to conclusively proof this role. The identity of the transporter(s) responsible for metal translocation across the arbuscule plasma membrane is also unknown, with candidates belonging to the metal eﬄux families indicated above.

Nutrient recovery from the periarbuscular space is mediated by transporters specific of infected cells for phosphate and ammonia (Javot et al., 2007; Guether et al., 2009). By analogy, it can be expected that metal transporters specific of the colonized cells are also mediating metal uptake. Transcriptomic analyses of these cells indicate that at least they specifically express a Ctr gene, and they up-regulate members of other metal uptake families (ZIP and Nramp; Gaude et al., 2011; Hogekamp and Küster, 2013). In maize, OPT8 could putatively be involved in iron recovery from the periarbuscular space, given that its expression is highly induced in mycorrhizal roots (Kobae et al., 2010). The role of this transporter is also supported by the co-upregulation of NAS genes (Kobae et al., 2010). Further detailed analyses of these proteins still remain to confirm this putative involvement, however, the already available data reflect an increased metal uptake at the cortical layers of mycorrhizal plants, consistent with a role of AMF in delivery metals to their host.

Although not as many as for arbuscular mycorrhiza, there are reports that indicate that ectomycorrhizal fungi are also able to buffer the plant from low or high metal levels (Bücking and Heyser, 1994; Langer et al., 2012). In these organisms, a vacuolar pathway is very likely used to deliver metals from soil to the host, as it is the case for K^+^ (Garcia et al., 2013). Once inside the root, within the hyphae of the Hartig net, metals and other nutrients will be delivered by a poorly characterized process.

## Metal Delivery to Sink Organs

Once the metals are incorporated from soil they are transported to sink organs where the demand is higher. Arguably, photosynthesis is the plant physiological process with the highest transition metal requirements, at least during vegetative growth (Yruela, 2013). Consequently, an important part of the transition metals recovered from soil are directed to leaves. In legumes there are also additional metal sinks, the root nodules. In these organs, symbiotic nitrogen fixation is carried out, which also has relatively large transition metal requirements (Brear et al., 2013; González-Guerrero et al., 2014). Transition elements are also important for plant reproduction (Sancenón et al., 2004; Roschzttardtz et al., 2011; Mary et al., 2015), and as the plant flowers and produces seeds, metals are redirected to these new organs.

### The Leaves

Once the metals are into the root cortex, either through the epidermis or through AMF, they symplastically and apoplastically reach the endodermis (**Figure 2**). Therefore, a number of metal transporters must exist to mediate metal release to the apoplast and subsequent apoplastic metal uptake and, at the pericycle, other metal transporters mediate their release into the xylem. However, there is not much information on transition metal transporters uploading or unloading metals to the apoplast. This is likely due to the lack of phenotype of mutants in genes that would play a role in endodermal transporters required for apoplast metal uptake. This seems to indicate that the symplastic metal translocation pathway is the most predominant or at least sufficient to satisfy most of the plant metal demands. In spite of this, localization studies of different metal transporters indicate that Zn^2+^ uptake from the root apoplast could be carried out by AtZIP2 in *A. thaliana* root endodermis (Milner et al., 2013). This is a Zn^2+^ uptake transporter that localizes mainly in the root stele. Nramp transporters could play a similar role. Recently, a *M. truncatula* iron transporter, MtNramp1, has been localized in the root endodermis (Tejada-Jiménez et al., 2015). When the encoding gene is knocked-out, neither growth alteration nor iron-deficiency phenotypes is observed, indicating that the apoplastic pathway for metal delivery to leaves is not essential. However, apolastic iron precipitates were observed in the root cortex, consistent with a role in apoplastic iron uptake.

More is known about how metals are released into the xylem. P-type ATPases play a role in metal extrusion into the xylem. In *A. thaliana*, HMA2 and HMA4 are the two Zn^2+^-ATPases that mediate long-distance Zn^2+^ transport (Eren and Argüello, 2004; Hussain et al., 2004), while HMA5 is likely involved in Cu^+^ translocation (Andrés-Colás et al., 2006). Iron and cobalt xylem loading is mediated by IRG/FPN transporters (Morrissey et al., 2009). However, at the pH present in the xylem, most metals tend to precipitate (Álvarez-Fernández et al., 2014). As a result, a number of metal binding molecules are present to allow for metal trafficking along the sap. In the case of iron, citrate seems to be the main responsible for maintaining metal solubility (Tiffin, 1970; López-Millán et al., 2000). This is evidenced by the detection of iron-citrate complexes in tomato xylem sap using high performance chromatographic methods (Rellán-Álvarez et al., 2010). Further support of the preeminent role of citrate as iron carrier in the xylem sap is the characterization of the *FRD3* gene in *Arabidopsis* encoding a citrate eﬄux protein (Rogers and Guerinot, 2002; Durrett et al., 2007). FRD3 mutation results in iron deficiency in shoots and an accumulation of this metal in the roots, consistent with a major reduction of root to shoot transport. This transporter is not only involved in xylem citrate loading, but more generally in iron transport across symplastically disconnected tissues (Roschzttardtz et al., 2011). Copper seems to be primarily associated to deoxymugenic acid in monocots, or to its precursor NA in dicots (Álvarez-Fernández et al., 2014). The identity of zinc speciation in the xylem sap still remains elusive, being histidine, NA, citrate, or cysteine candidate molecules (Álvarez-Fernández et al., 2014).

**FIGURE 3:**
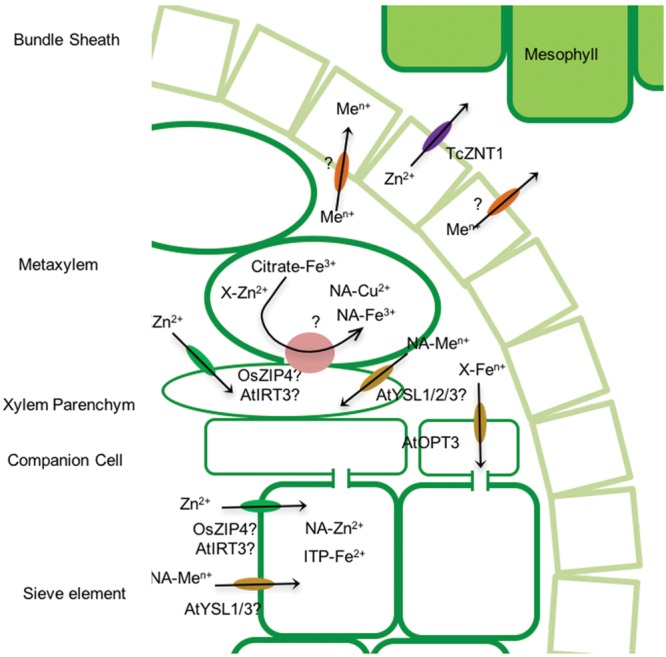
**Metal delivery to the mesophyll cells.** At is used as a prefix to indicate *A. thaliana* proteins, Os for *Oryza sativa*, and Tc for *Thlaspi caerulescens*. X is used to indicate an unknown metal chelator. Me^n+^ stands for a general metal.

Metal release from the xylem is not very well characterized. It has to go from the xylem into the mesophyll cells (**Figure 3**). Based on their expression pattern, ZIP transporters AtIRT3 and OsZIP4 might be involved in this process in the case of zinc (Olsen and Palmgren, 2014). TcZNT1 could be responsible also for zinc extrusion from the vasculature (Küpper and Kochian, 2010). In addition, YSL transporters would also be involved in metal unloading from the xylem (Conte and Walker, 2011), as indicated by the study of the tomato mutant *chloronerva.* This mutant shows intervenal chlorosis, the result of the mutation of gene encoding for a NA synthase gene (Ling et al., 1999). Due to this mutation, iron unloading from the xylem is impaired causing the chlorosis. Consequently, at this level iron speciation must change from iron-citrate to iron-NA, suggesting that YSL transporters would be mediating iron transfer to the xylem. This is supported by the expression pattern of *AtYSL1, AtYSL2*, and *AtYSL3*, with a maximum xylem-associated parenchyma cells, and the phenotype of the *ysl1ysl3* mutant (DiDonato et al., 2004; Waters et al., 2006). Given the abundance of NA in these tissues (Stephan et al., 1990), and its affinity for multiple metals (Anderegg and Ripperger, 1989; von Wiren et al., 1999), it could be speculated that YSL transporters with different affinities for the different metal-NA complexes would be responsible for metal transfer from the xylem to the leaves.

### The Legume Nodules

Most legumes, in addition to the shoot, have other metal sinks: the root nodules. These are differentiated root organs that develop as the result of the interaction with specific soil bacteria species generally known as rhizobia (*Rhizobium, Bradyrhizobium*,…) (Elkan, 1981; Oldroyd, 2013; Downie, 2014). Rhizobia, upon detecting specific flavonoids released by the host plant, produce specific nod factors, that are detected by proteins of the LysM-receptor kinase family in the root epidermis (Oldroyd, 2013; Downie, 2014). This triggers a signaling cascade that results in curling of a root hair, invagination of the plasma membrane, that guides the rhizobia trapped by the root hair into the plant. As this is occurring, cells dedifferentiate in the root pericycle and develop a nodule primordia, in a process with many common aspects to lateral root development (Xiao et al., 2014). Rhizobia reach the nodule cortex, and are released into the host cell cytosol, in an endocytic-like process (Huisman et al., 2012). There, in the appropriate biochemical conditions, rhizobia differentiate into bacteroids (Kondorosi et al., 2013), surrounded by a plant-derived membrane, resulting in the symbiosomes (Clarke et al., 2014). Nodulation is a process that shares many common elements with mycorrhization (the chemical nature of nod and myc factors, or the common signaling pathway), as it very likely evolved from it (Parniske, 2008; Gutjahr and Parniske, 2013; Oldroyd, 2013).

In the symbiosomes N_2_ is converted into NH_4_^+^, a process catalyzed by the enzyme nitrogenase. This enzyme represents 10% of the total bacteroid proteins and has a unique iron-molybdenum cofactor (FeMoco) that with the assistance of two other Fe-S clusters is responsible for the reduction reaction (Miller et al., 1993). This process requires large amounts of energy and is very sensitive to O_2_, which poisons the enzyme. However, rhizobia are strict aerobes and cannot produce energy in anaerobic conditions. For this reason, the enzyme leghemoglobin (20% of the nodule protein) is critical for the reaction, since it is able to control O_2_ levels in the nodule (Appleby, 1984). It has a high affinity Fe-heme cofactor that keeps O_2_ concentrations below 1 μM (Soupène et al., 1995). This high affinity also requires the existence of high affinity cytochrome oxidases that can use O_2_ as electron acceptor when present at very low concentrations. This is cytochrome oxidase cbb3 that has a iron and copper cofactors (Preisig et al., 1996b). Other metalloenzymes are also critical for symbiotic nitrogen fixation, such as many of the detoxifiers of the free radicals that are produced in the nodule (Becana et al., 2010). The variety of metalloproteins and the high concentrations required of them, make the nodule one of the main metal sinks in legumes. In fact, metal bioavailability limits nodule appearance and development (Tang et al., 1991, 1992). To avoid this, nodulated plants typically induce their metal deficiency responses to ensure an adequate supply of metals to the nodule (Terry et al., 1991). This response also indicates that all these metals have to be provided by the host plant, rather than using any type of rhizobial metal reservoir.

**FIGURE 4:**
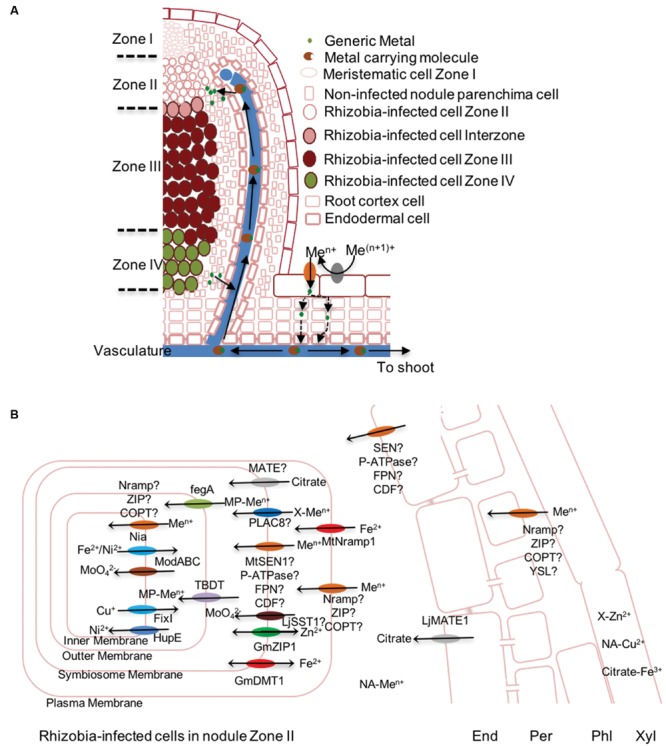
**Metal transport in indeterminate nodules. (A)** General overview of metal delivery and recovery to legume nodules. **(B)** Detail of transport process to deliver metals to symbiosomes in the nodule Zone II. Mt is used as a prefix to indicate *M. truncatula* proteins, Gm for *G. max*, and Lj for *Lotus japonicus*. X or MP are used to indicate an unkown metal chelator or a general metallophore, respectively. End stands for Endodermis, Per for Pericycle, Xyl for Xylem, Phl for Phloem, and Me^n+^ for a general metal.

Metal delivery to the nodule could, theoretically be through the epidermal layer (as in roots), delivered by the vasculature (as the shoot), or use pre-existing metal reserves. Studies of metal visualization using synchrotron based X-ray fluorescence in *M. truncatula* indeterminate type nodules indicate that iron, and likely the other elements, are delivered by the vasculature (Rodríguez-Haas et al., 2013). Indeterminate nodules are a type of nodule that has an apical meristem, and is, in theory, able to grow indefinitely. As it does, different developmental zones appear. Zone I is the meristematic region, Zone II is the zone where rhizobia reach the cells and differentiate into bacteroids, Zone III is the fixation zone, and Zone IV is the senescent one (Vasse et al., 1990). To this, some authors add the interzone, between Zones II and III, and Zone V, where the rhizobia act as saprobes (Timmers et al., 2000; Roux et al., 2014). By studying metal distribution along an indeterminate nodule, S-XRF analyses could study the different stages of nodule development. The results showed no metal storages either in the meristematic region or in the epidermis. In Zone II, iron accumulated in the apoplast, while in Zone III it was located in the infected cells, associated to symbiosomes. In Zone IV, the metal concentration in the infected cells diminished and they were relocalized to the vasculature. These observations were consistent with a model in which the metals were primarily delivered by the vasculature (**Figure 4A**) (Rodríguez-Haas et al., 2013). Ferroreductase activity in nodules is also increased (Slatni et al., 2011), what could indicate either that there is also some input from the nodule epidermis, or that metals have to be reduced within the nodule. The existence of a Fe^2+^ transporter in rhizobia infected nodule cells in zone II (Tejada-Jiménez et al., 2015), and the very likely use of citrate as an Fe^3+^ carrier to the nodule (Takanashi et al., 2013), would indicate that the induction of nodule ferroreductase activity observed is to facilitate metal upload by rhizobia-infected cells. No S-XRF analyses have been carried out in determinate-type nodules (as in soybean, without the meristematic region, and consequently, no clear zonation). However, the mutation of a nodule-specific citrate transporter (*LjMATE1*) results in iron accumulation in the vasculature and reduced nitrogen fixation rates (Takanashi et al., 2013), indicating that metal delivery in determinate type nodules would also be through the vessels.

Once in the apoplast of Zone II, metals have to be incorporated by the cells (**Figure 4B**). It is to be expected that members of the ZIP, Nramp, YSL, and Ctr families are responsible for this. A *M. truncatula* Nramp transporter, MtNramp1, has been show to play a role in iron uptake by cells in Zone II (Tejada-Jiménez et al., 2015). This transporter is localized in the plasma membrane of these cells, and *nramp1* mutant plants have reduced nitrogen fixation capabilities which are restored when the mutated gene is reintroduced or when the plants are watered with iron-fortified solutions. Zone II is the only region of the nodule where *MtNramp1* is expressed, supporting the hypothesis that this area is where metals are released form the vessels. Nramp transporters can also transport other divalent metals, and consequently MtNramp1 could conceivably introduce other elements in the cells. However, no changes in the concentration of other elements where observed in either nodules or roots of *nramp1* plants. Moreover, nitrogenase activity in these mutant lines was only reduced 60%, what indicates that iron must be uploaded through other systems, perhaps a yet-to-be-determined ZIP transporter.

In addition to citrate, NA should also be playing an important role in metal speciation in the nodule, since mutation in a NA synthase gene in *M. truncatula* results in a loss of nitrogenase activity in nodules (Avenhaus et al., 2016). This result indicates that during nodule maturation, metal speciation changes with an important effect on the functioning of the symbiosomes. It could be due to several different possibilities, such as NA being the intracellular metal carrier, NA being the metal donor for a specific intracellular transporter required for nitrogen fixation in the nodule, or NA mediating metal delivery to the nodule. More information on nodule metal speciation and in the specific localization of each metal species is required to draw further conclusions.

After being introduced into the rhizobia-infected cells, cytosolic metals have to be delivered to the same organelle as in a regular plant cell, and to symbiosomes. There is very little information on how this is done. It could merely rely in mass action effects and that metals are delivered to the organelle that has a bigger demand, and consequently a bigger “pull” on the metal reserves. Other possibility is a more directed way, either by establishing different pools accessible to only some acceptor, or by using different metal carrying proteins depending on its final destination. Identifying this mechanism is critical toward the current efforts to develop nitrogen fixing capabilities in non-legumes, since providing the metal cofactor in a timely manner is essential for nitrogenase assembly and function. These efforts will be greatly helped by the unequivocal identification of the proteins required for metal transport across the symbiosome. Seminal work by Moreau et al. (2001) and Kaiser et al. (2003) identified in *Glycine max* a ZIP and a Nramp transporter that were connected to metal transfer to the symbiosomes. However, the transporters belonging to these families that have been biochemically characterized transport metals toward the cytosol (**Figure 1**) (Nevo and Nelson, 2006; Lin et al., 2010). As a result, it could be speculated that GmZIP1 and GmDMT1 could more likely be involved in preventing metal overload of the symbiosomes by facilitating metal eﬄux from them. More recently, Hakoyama et al. (2012) have identified a *sen1* mutant in *G. max* and in *M. truncatula* that is affected in symbiotic nitrogen fixation. *SEN1* encodes a nodule-specific protein belonging to the VIT1/CCC1 family, which are located in organelle, transporting iron into their lumen. This would make SEN1 a good candidate to directly provide metal through the symbiosome membrane, although information of its subcellular localization has not been provided yet. It would also be expected a MATE transporter collaborating in iron transport across the symbiosome membrane. Guerinot et al. (1990) showed that *B. japonicum* prefers citrate as the chelator for iron uptake in free living conditions. If this is also valid for the nodule, we would expect a citrate-exporter, similar to *A. thaliana* FRD3, that would translocate citrate into the peribacteroid space. Zinc transport into the symbiosome could also potentially be carried out by proteins carrying he PLAC8 superfamily motif recently identified in the proteome of *G. max* nodules (Clarke et al., 2015), some of which have been suggested to play a role in zinc transport (Song et al., 2010a). In the case of copper, it would be expected that a P_1b_-ATPases would play this role. However, in the available transcriptomic data there is no ATPase upregulated in the nodule (Benedito et al., 2008; Roux et al., 2014). Similarly to copper, no molybdate transporter has been identified in the symbiosome membrane in spite of the importance of molybdenum in symbiotic nitrogen fixation. However, some sulfate transporters can also mediate molybdate transfer across membranes (Fitzpatrick et al., 2008). Consequently, symbiosome-specific sulfate transporter SST1 (Krussell et al., 2005) could also facilitate molybdenum delivery to bacteroids.

Once they cross the symbiosome membrane, metals are accumulated in the peribacteroideal space, as indicated by radiotracer studies of iron (LeVier et al., 1996). From there, metals have to cross the rhizobial outer membrane. Very little information is available on the identity of these metal transporters. Based on the study of metal uptake by free-living rhizobia or by pathogenic bacteria, it can be speculated that a metallophore-mediated system could be used (LeVier et al., 1996; Postle and Larsen, 2007; Aznar et al., 2014). Many of these complexes are substrate of the TonB-dependent transporters (TBDTs; Postle and Larsen, 2007), that can mediate the uptake of iron, zinc, cobalt, or nickel complexes (Chakraborty et al., 2007; Noinaj et al., 2010). Further support for metallophore role in metal transport across bacteroideal outer membrane is that *R. leguminosarum* vicibactin receptor is induced both in free-living bacteria grown under iron-limiting conditions, and in the infection zone of pea nodules (Yeoman et al., 2000), the area where metals are likely incorporated (Rodríguez-Haas et al., 2013). However, other systems are probably in place, since mutation of these transport systems does not show any major effect on symbiotic nitrogen fixation capabilities (Yeoman et al., 2000; Lynch et al., 2001).

From the bacteroideal periplasm, metals have to be transported into the cytosol. Molybdenum is introduced as a molybdate anion by the ModABC system (Delgado et al., 2006; Cheng et al., 2016). This transport complex is essential for nitrogenase maturation. Those rhizobia that also express hydrogenase require Ni^2+^ uptake, which is mediated by the transporter HupE (Brito et al., 2010). The identity of the transporters of other essential metals (iron, copper, zinc,…) has not been determined, what indicated that this might be a robust process where the mutation of single transport genes might not be enough to substantially reduce their transport. Metal eﬄux is also a very important process in bacteroids both to avoid metal toxicity and to metallate periplasmic proteins. In some cases, metal delivery by the plant systems could theoretically overload the bacterial metal homeostatic mechanisms, as seems to be indicated by the presence of a metal pool in the peribacteroideal space (LeVier et al., 1996). In this sense, bacteroids express P-type ATPases, CopA1 for Cu^+^, and Nia1 for Fe^2+^ and Ni^2+^ in the case of *S. meliloti* (Zielazinski et al., 2013; Patel et al., 2014). These systems are not critical for symbiotic nitrogen fixation, although, they are expressed in symbiotic conditions and they confer metal tolerance under free-living conditions. Another Cu^+^-ATPase, FixI is responsible for providing the Cu^+^ cofactor to high-affinity cytochrome oxidases (Preisig et al., 1996a). In some rhizobia, there has been a duplication of *fixI*, resulting in differential roles along the nodule (Patel et al., 2014). In addition, there is a third type of Cu^+^-ATPase, CopA3, which would be responsible for metallating some of the cuproenzymes involved in resisting the initial assault of the plant immune system in the early stages of nodulation (Patel et al., 2014).

Similar mechanisms of metal transport must be in place for other endosymbiotic interactions in which the microsymbiont is isolated from soil, as is the case of actinorhiza. In this sense, it has been shown that nodulated *Alnus glutinosa* plants allocate more molybdenum to roots (Bélanger et al., 2013; Pourhassan et al., 2015), consistent with the synthesis of FeMoCo for nitrogen fixation. In fact, control of nutrient delivery would be a possible mechanism of controlling the microbiont proliferation, since by limiting access to essential nutrients or encouraging it, growth rates can be modulated. However, very little is known on the specifics of modulation of metal transfer to other beneficial endosymbiotic bacteria.

### The Seeds

Metal delivery to seeds and to younger leaves seem to be carried out through the phloem (Curie et al., 2008). At nodes in the stem and minor veins metal are very likely transferred from the xylem to the phloem (Andriunas et al., 2013). In the case of iron and *A. thaliana* this seems to be mediated by an OPT transporter, OPT3 (**Figure 3**) (Zhai et al., 2014). This transporter is localized in companion cells in minor veins and stem nodes, and its mutation results in iron accumulation in the vicinity of these veins, as well as in the xylem sap. As expected of a OPT transporter, it does not use iron as substrate but rather an iron-chelator complex to be determined. No information is available for how other transition elements are transferred from the xylem to the phloem.

In addition to transfer from the xylem, phloem also obtains metals from senescent organs. As the plant flowers, sink organs such as leaves and nodules (in the case of legumes) senesce and their nutrients are recycled. Alterations in NA levels results in reproductive abnormalities, indicating that this molecule participates in metal delivery to the flowers (Stephan et al., 1990; Takahashi et al., 2003). Consistent with this is the role of YSL transporters in metal seed loading. Expression of *AtYSL1* and *AtYSL3* in *A. thaliana* peaked during leaf senescence (Waters et al., 2006). Moreover, double mutant *ysl1ysl3* plants had higher zinc and copper content in leaves, while reduced levels of iron, zinc, and copper where detected in their seeds. In rice, OsYSL2 and OsYSL16 would have a similar function (Ishimaru et al., 2010; Zheng et al., 2012). Legumes also recycle their metals from the nodules (Burton et al., 1998), very likely mediated by NA, as indicated by the identification of a NA synthase gene specific of senescent nodules that it is expressed in the vasculature (Hakoyama et al., 2009), and by the iron relocation observed in the senescent areas of nodules (Rodríguez-Haas et al., 2013). Overall, these and other data suggest that senescent organs are an important source of metals for flowering and embryo development (Hocking and Pate, 1977; Burton et al., 1998).

Although NA plays an important role in phloem loading, it does not seem to be the major iron chelating agent in it (Álvarez-Fernández et al., 2014). Iron is mostly associated to high molecular weight molecules, such as the iron transport protein ITP discovered in *Ricinus communis* (Krüger et al., 2002). In contrast zinc and copper seem to be mostly associated to NA (Álvarez-Fernández et al., 2014), although some complexes with higher molecular-weight molecules such as metallothioneins or metallochaperones can also be detected (Mira et al., 2001; Lattanzio et al., 2013).

Metals are also required for gametogenesis. Copper is needed for pollen tube development, as indicated by pollen abnormalities detected in *copt1 A. thaliana* plants (Sancenón et al., 2004). Iron, delivered as iron-citrate with the help of FRD3, is also essential for pollen development (Roschzttardtz et al., 2011). FRD3 could also play a role in embryogenesis, as citrate would solubilize the iron in the nutritive solution around the developing embryo (Roschzttardtz et al., 2011), although some other transporter ought to be involved since no developmental alteration was observed in *frd3* embryos. Iron delivered to the embryos is directed to vascular tissues were it will be stored in vacuoles in endodermal cells (Roschzttardtz et al., 2009). In *A. thaliana*, AtVIT1 seems to be mediating the last step, introducing iron in these vacuoles (Kim et al., 2006). Upon germination, Nramp3 and Nramp4 could be responsible for remobilization of these iron reserves, as iron is stored in the vacuoles of endospermal cells to be later used as the seed germinates (Lanquar et al., 2005).

## Conclusion

In the last three decades, we have gained a deep insight on what transporters are involved in root metal uptake and translocation to the shoot. We have also identified many of the metal-carrier molecules, as well as unveiled many of the complex regulatory pathways. More recently, as technology improved, the role of microbes in plant metal homeostasis is being better understood, as are the mechanisms mediating metal exchange with the endosymbionts. However, several other aspects have been insufficiently addressed. For instance, information on metal homeostasis in other, less studied, endosymbiosis is still lacking, very probably due to the difficulties of obtaining axenic cultures for some of them. More information is also required of the microbiome of plants growing under different levels of metal nutrition. In addition, although we know of multiple different metal transporters and some carrier proteins, their final destination, the identity of the metalloproteins that will use these metals, remains elusive. Consequently, we are missing a key element to better understand intracellular metal trafficking and use. At a systemic level we still need to determine which are the metal sensors, the signals that determine the plant metal nutritional levels, as well as to determine how the plant controls the shoot to root metal fluxes. This later aspect is especially important in legumes, since symbiotic nitrogen fixation is also an important metal sink and metal partitioning with leaves is critical to correctly balancing carbon and nitrogen fixation rates. Steps are being taken to tackle this question in the coming years. Improved methods for metalloproteomics are being developed, and elements involved in shoot-to-root metal transport in legumes are being unveiled to have a better understanding of metal partitioning in legumes, which together with improved metal imaging and metal speciation methods point toward obtaining a very clear picture on how plants use metals and the role that microorganisms have on plant metal homeostasis. This will allow us to select inoculants which will improve plant metal uptake, as well as cultivars with enhanced metal recovery capabilities from AMF or from senescent nodules, as well as increased delivery for symbiotic nitrogen fixation.

## Author Contributions

VE wrote the section on plant metal uptake, ÁS the section on metal transport in the nodule, and MT-J the section on arbuscular mycorrhiza and the molybdenum transport sections. MG-G outlined the manuscript, wrote the remaining sections, and put together the manuscript.

## Conflict of Interest Statement

The authors declare that the research was conducted in the absence of any commercial or financial relationships that could be construed as a potential conflict of interest.
